# “Our voices matter”: a before-after assessment of the effect of a community-participatory intervention to promote uptake of maternal and child health services in Kwale, Kenya

**DOI:** 10.1186/s12913-018-3739-9

**Published:** 2018-12-04

**Authors:** Vernon Mochache, Eunice Irungu, Hajara El-Busaidy, Marleen Temmerman, Peter Gichangi

**Affiliations:** 1 International Centre for Reproductive Health, Mombasa, Kenya; 2 University of Ghent, Ghent, Belgium; 3 County Government of Kwale, Department of Health, Kwale, Kenya; 4 Aga Khan University, Nairobi, Kenya; 5 University of Nairobi, Nairobi, Kenya

**Keywords:** Dialogue model, Community-participatory approaches, Family planning, Antenatal care, Facility-based delivery, Kwale, Kenya

## Abstract

**Background:**

Community-participatory approaches are important for effective maternal and child health interventions. A community-participatory intervention (the Dialogue Model) was implemented in Kwale County, Kenya to enhance uptake of select maternal and child health services among women of reproductive age.

**Methods:**

Community volunteers were trained to facilitate Dialogue Model sessions in community units associated with intervention health facilities in Matuga, Kwale. Selection of intervention facilities was purposive based on those that had an active community unit in existence. For each facility, uptake of family planning, antenatal care and facility-based delivery as reported in the District Health Information System (DHIS)-2 was compared pre- (October 2012 – September 2013) versus post- (January – December 2016) intervention implementation using a paired sample t-test.

**Results:**

Between October 2013 and December 2015, a total of 570 Dialogue Model sessions were held in 12 community units associated with 10 intervention facilities. The median [interquartile range (IQR)] number of sessions per month per facility was 2 (1–3). Overall, these facilities reported 15, 2 and 74% increase in uptake of family planning, antenatal care and facility-based deliveries, respectively. This was statistically significant for family planning pre- (Mean (M) = 1014; Standard deviation (SD) = 381) versus post- (M = 1163; SD = 400); t (18) = − 0.603, *P* = 0.04) as well as facility-based deliveries pre- (M = 185; SD = 216) versus post- (M = 323; SD = 384); t (18) = − 0.698, *P* = 0.03).

**Conclusions:**

A structured, community-participatory intervention enhanced uptake of family planning services and facility-based deliveries in a rural Kenyan setting. This approach is useful in addressing demand-side factors by providing communities with a stake in influencing their health outcomes.

**Electronic supplementary material:**

The online version of this article (10.1186/s12913-018-3739-9) contains supplementary material, which is available to authorized users.

## Background

Slow progress on the Millennium Development Goals redirected focus towards addressing the Primary Health Care ideals laid out in the Alma Ata Declaration of 1978 [1–6]. Key amongst these was the involvement of communities in the planning and implementation of health interventions targeting them. Such a participatory approach is essential for negotiated decision-making, shared commitment, social accountability, enhanced ownership and ultimately, assured sustainability of these interventions [7, 8]. For this reason, the World Health Organization currently recommends active community participation during the development and implementation of interventions aimed at specifically improving maternal and child health (MCH) outcomes [9].

For MCH services in particular, community participation is an important component of a human rights-based approach to promoting health and well-being [10]. It emphasizes the need to take into consideration patterns of individual behavior that could ultimately affect uptake of health services. It also seeks to address inherent structural determinants of health outside of the formal health system, including socio-cultural factors, which could ultimately influence the health-seeking behavior of individuals within a particular community [11–15].

There is an increasing appreciation of the importance of addressing demand-side factors to improve uptake and utilization of MCH services [16, 17]. Addressing factors that influence demand for these services requires the development of interventions that aim to be not just effective, but also locally responsive and culturally appropriate [18, 19]. Such interventions are anchored on the understanding that consumers of health services, be they individuals or communities, ought to be partners in improving the delivery of these services and ultimately, enhancing health outcomes [20]. As a result, it is important that they participate in the design, planning and implementation of health interventions targeting them to ensure their buy-in and assure future sustainability.

The Dialogue Model (DM) is a structured, community-participatory intervention that is anchored on the critical pedagogy theory advocated by the Brazilian educator/philosopher Paulo Freire [21–23]. It utilizes problem-posing techniques to stimulate societal reflection and raise critical awareness (*conscientization*) of the situation to provoke reflective action. Ultimately, it seeks to promote a deeper understanding of local factors that lead communities, households and individuals to seek to remain healthy so that external interventions can be effective [24].

This approach recognizes the central role that communities play in individual decision-making towards positive behaviour change and leverages on social capital i.e. the networks developed within communities that are intended to achieve common good based on trust, cooperation and reciprocity. Social capital assumes that individual behaviour change is driven by the desire for seeking validation and belonging within a wider community. As a result, an individual is likely to adopt behaviors that endear them to their wider social network [25, 26]. On the other hand, wider community actions are assumed to work in the best interests of individuals in that society. As such, these community actions are more likely to be adopted by individuals towards the wider common good.

A DM approach is especially relevant in parts of the developing world where uptake and utilization of critical health services has been sub-optimal [27]. In these settings, there is usually a power dynamic at play between health care workers (HCWs) on one hand, and consumers of health services on the other. The HCWs are typically considered dominant purveyors of health information while patients are expected to remain as passive recipients. As a result, HCWs often assume that what they have advised has been heard, understood, accepted and will be done. Unfortunately, this is never the case in many instances [28]. This approach therefore, provides consumers of health services with a stake in determining how their health should be managed. It has previously been used to promote various social change initiatives in health and development [29, 30].

Kwale County in coastal Kenya consists of residents who are predominantly rural (20% urbanization), Muslim (80%), from the Digo community (80%) with a very high poverty rate (75%). The 2014 Kenya Demographic and Health Survey (KDHS) had previously revealed a high total fertility rate of 4.7, low family planning (FP) utilization with a contraceptive prevalence rate (CPR) of 42% and high unmet need at 21% in this setting [31]. Additionally, only 49% of women reported having delivered in a health facility. Just like the national average, 96% of women reported having received antenatal care (ANC) during their last delivery although only ~ 60% of these reported having made the recommended ≥4 ANC visits. These findings reflect a slight improvement over time [32–36].

With this background, the DM intervention was implemented in Matuga sub-county, Kwale with the aim of promoting uptake of select MCH services specifically, FP, ANC and facility-based delivery. It consisted of regular DM sessions in community units (CUs) linked to health facilities where an ongoing, multi-country, operational research project (the Missed Opportunities in Maternal and Infant (MOMI) health) was being implemented. The broad objective of the MOMI project was to reduce maternal and infant mortality through implementing a set of context-specific interventions combining facility and community-based strategies [37].

### Study objectives and AIMS

The overall objective of this study was to determine the effect of implementing a structured, community-participatory intervention (the Dialogue Model) on the uptake of select MCH services. Specifically, the study aimed:

- To determine whether conducting regular DM sessions would increase the uptake of FP, ANC and facility-based delivery in facilities associated with CUs where the sessions were implemented
- To develop recommendations for improving uptake and utilization of MCH services in this setting using structured, community-participatory approaches

## Methods

### Study setting and design

Community units (CUs) are established as part of the Community Health Strategy of Kenya’s Ministry of Health (MoH). Each CU comprises of ~ 1000 households and is aligned to official administrative sub-units (sub-locations) comprising of several villages. Each CU is served by ~ 50 community health volunteers (CHVs) i.e. each CHV serves ~ 20 households and is supervised by a community health extension worker (CHEW) who is typically an HCW from the primary care facility to which the CU is linked. At the time of implementing the current study, the County Government of Kwale had adopted the MoH’s Community Health Strategy and prioritized setting up of CUs for high-volume facilities serving large catchment populations.

The DM intervention was implemented between October 2013 and December 2015 nested within the framework of the MOMI project that was funded by the European Commission Seventh Framework Programme (Grant Agreement #265448). This project was implemented in 10/20 (50%) facilities in Matuga sub-county and their associated CUs (intervention facilities) and included interventions at multiple levels including the county health administration, health facility as well as community. The intervention facilities were selected purposively as they were the only ones that had active CUs at the time i.e. CUs with a clearly-mapped geographic scope and CHVs selected and trained as per the MoH’s guidelines.

As a result of the Kenyan government’s policy of free maternity services enacted in early 2013, the bulk of rural dispensaries in Kenya established maternity delivery units [38, 39]. These units enabled pregnant mothers to access delivery services at primary care level. Complicated deliveries are typically referred to more specialized levels for advanced care. Delivery units at lower levels are typically manned by a nurse-midwife and consist of 1–2 delivery beds. Additionally, all pregnant women in Kenya receive ANC follow up at primary care level including any recommended prophylaxis and supplementation.

### Intervention implementation

The DM sessions followed a series of standardized steps as outlined in the study-specific procedures developed a priori to guide the organization and conduct of each session (Additional file 1). The procedures required that local CHVs mobilize participants from their communities to attend sessions disaggregated by age and gender. These CHVs also selected a date and venue for the session and informed the local administrator (chief/village elder) as well as an HCW from the local facility who would be present during the session to clarify any health-related issues. Since DM sessions were meant to be held at the convenience of community members, no specific number was planned from the onset. The CHVs were encouraged to convene sessions as regularly as practicable aiming to conduct at least one session per month in their community.

During the session, a local community member, typically a CHV chosen to suit the age and gender of the session’s participants and who had prior training on effective conduct of a DM session, would act as session moderator. Prior training for moderators focused on encouraging use of open-ended and probing questions, conducting the session using techniques that affirmed each participant’s contribution and promoting reflective listening with paraphrasing of each participant’s contributions. Moderators were also trained to remain neutral and ensure that they maintained group control so that that some participants do not dominate while encouraging silent ones to engage in the discussion.

Each DM session was initiated using a dialogue stimulator/starter, in our case, an informational picture booklet. The purpose of this starter was to stimulate initial discussions focusing on the issues targeted for deliberation. Specifically, the issues discussed during the sessions revolved around promoting uptake and utilization of FP, ANC and facility-based deliveries, including discussions around barriers and facilitators to uptake and how to effectively deal with these as a community and individuals. The informational picture booklet was simple, specific, culturally sensitive, posing a single problem without providing a solution and adapted to the audience’s age and gender.

The session moderator then posed a series of questions that aimed to identify and define the issues and confirm relevance to session participants. For example, “What did you see in the pictures? Did you identify a health problem? What was the problem? Does this problem occur in this community?” Participants then proceeded to provide individual testimonies of actual experiences with the issues identified. This step was also meant to get session participants to start talking and enabled them to define the issues under consideration from their own perspective and to emotionally own the problem as well as begin to reflect on any needed improvement.

The next step in the session was meant to identify current actions to addressing the issues identified and the extent to which they could achieve desired results. The question posed was “Why does the issue identified persist despite current efforts?” This step was meant to promote an analysis of the causes of the issue and develop consensus that the current situation could be improved. This step was also meant to identify new actions/options necessary to solve the issue from the perspective of the community. Through brainstorming, a list of actions was generated and appraised in terms of effectiveness and feasibility.

The final step involved generating commitment by participants to consider and list the consequences of taking or not taking the recommended actions. The question posed was “What do you think will be the results of carrying out the recommended action?” Having confirmed the importance and urgency of actions to be taken, session participants then proceeded to prepare an action plan detailing what will be done, by whom, when and with what resources. For each session, a facilitator, typically another CHV, kept a record of issues that were discussed and the agreed upon action plan. They also completed a session event log and shared this with study investigators who provided regular supportive supervision in conjunction with county/sub-county health administrators.

### Facilitation of CHV activities

Community health volunteers were provided with training on how to effectively conduct a DM session. This was an adaptation of the CHV training curriculum offered by the Kenyan MoH and incorporated aspects of the standardized DM procedures. The CHVs did not receive any monetary payment for their services. Instead, they were reimbursed for travel and meals when they attended trainings. They were also trained on how to organize themselves into informal community self-help groups for income generation. The trainings lasted a week at a time and were meant to improve the capacity of CHVs to effectively conduct their roles as well as to promote an avenue for continued self-sustenance. The local CHEW supervised CHVs’ activities and each provided monthly written reports of their activities.

### Sample size and sampling procedures

The 12 CUs associated with the 10 intervention facilities where DM sessions were held were sampled purposively as they were the only ones in Matuga sub-county at the time of implementing the MOMI project that were active. Depending on geographic scope, each CU covered several villages. Villages where DM sessions were held were selected at the convenience of the CHVs organizing the meeting. Participants during the sessions were also sampled purposively according to the required age and gender. Separate sessions were held by age and gender to ensure cultural appropriateness and promote effective discussions. The total number of participants per DM session was restricted to 40 and each lasted up to 30 min.

### Community engagement and ethical considerations

In order to obtain buy-in, a series of meetings was held with community gatekeepers (religious leaders and local administrators) in collaboration with county/sub-county health management teams and other stakeholders prior to and during intervention implementation. Ethical approval for the study was obtained from the Ethics Review Committee of the University of Nairobi and Kenyatta National Hospital (P151/03/2014). A research permit was also obtained from the National Commission for Science, Technology and Innovation (#4703). Participants in the DM sessions provided group, oral informed consent.

### Data management and statistical analyses

Data on the number of DM sessions held per month was logged into a Microsoft Excel (2010) spreadsheet (Microsoft Inc. Seattle, WA, USA). Continuous data on the outcomes of interest were then abstracted per facility from the District Health Information System (DHIS)-2. Outcomes of interest included uptake of FP, ANC and facility-based deliveries with specific DHIS-2 indicators abstracted being: 1) number of women of reproductive age (WRA) receiving FP commodities, 2) number of new ANC attendees and 3) total number of deliveries. These were compared pre- (October 2012 – September 2013) versus post- (January – December 2016) intervention implementation using a paired sample t-test. All statistical analyses were conducted in Microsoft Excel (Microsoft Inc. Seattle, WA, USA) and all statistical tests were evaluated using an α-value of 0.05.

## Results

Between October 2013 and December 2015, a total of 570 DM sessions were held in 12 CUs associated with 10 intervention facilities in Matuga sub-county, Kwale. In the 2013/14 annual work plan, these facilities were estimated to have a total catchment population of 120,574 out of which 27,732 (23%) constituted WRA (Table 1). Of these, 1 was a district/county referral hospital (Level 4), 2 were health centers (Level 3) while 7 were dispensaries (Level 2). The 10 remaining facilities that did not receive the intervention comprised of 1 health center and 9 dispensaries and were estimated to serve a total catchment population of 60,966 out of which 14,021 (23%) were WRA.

**Table 1:** Characteristics of intervention and non-intervention health facilities in Matuga sub-county, Kwale

	Annual Work Plan 2013/2014				Annual Work Plan 2015/2016			
Health facility name	Total catchment population	Women of reproductive age (15–49 years)	Number of maternity/delivery beds	Number of nurse/midwives	Total catchment population	Women of reproductive age (15–49 years)	Number of maternity/delivery beds	Number of nurse/midwives
Intervention Health Facilities								
Kwale District Hospital	18,905	4348	10	25	20,017	4606	15	30
Tiwi Rural Health Centre	16,274	3743	4	10	17,232	3963	6	13
Mkongani Health Centre	24,108	5545	3	4	21,224	4882	5	8
Kizibe Dispensary	13,066	3005	1	2	13,835	3182	1	2
Magodzoni Dispensary	9395	2161	1	2	9948	2288	2	2
Matuga Dispensary	5550	1277	1	2	5877	1352	1	2
Mazumalume Dispensary	7313	1682	1	2	7743	1781	1	2
Mwaluphamba Dispensary	14,054	3232	1	3	14,881	3423	1	3
Vyongwani Dispensary	3209	738	1	2	3398	781	2	2
Ng’ombeni Dispensary	8700	2001	1	2	9212	2119	1	2
Sub-total	120,574	27,732			123,367	28,377		
Non-intervention Health Facilities								
Shimba Hills Health Center	6500	1495	2	2	6882	1583	3	4
Msulwa Dispensary	3556	818	1	1	3765	866	1	1
Mwapala Dispensary	5985	1377	1	2	6337	1458	2	2
Lukore Dispensary	4271	982	1	2	4522	1040	2	2
Kiteje Dispensary	5724	1317	1	2	6061	1394	2	2
Mkundi Dispensary	4721	1084	1	2	4989	1148	1	2
Kibuyuni Dispensary	4423	1017	1	1	4683	1077	1	1
Mwaluvanga Dispensary	4481	1031	1	1	4745	1091	1	1
Mbuguni Dispensary	3353	771	1	1	3550	817	1	1
Waa Dispensary	11,967	2752	1	2	12,671	2914	2	4
Sub-total	60,966	14,021			64,542	14,846		
Grand-total	175,546	40,376			187,909	43,223		

**Fig. 1:**
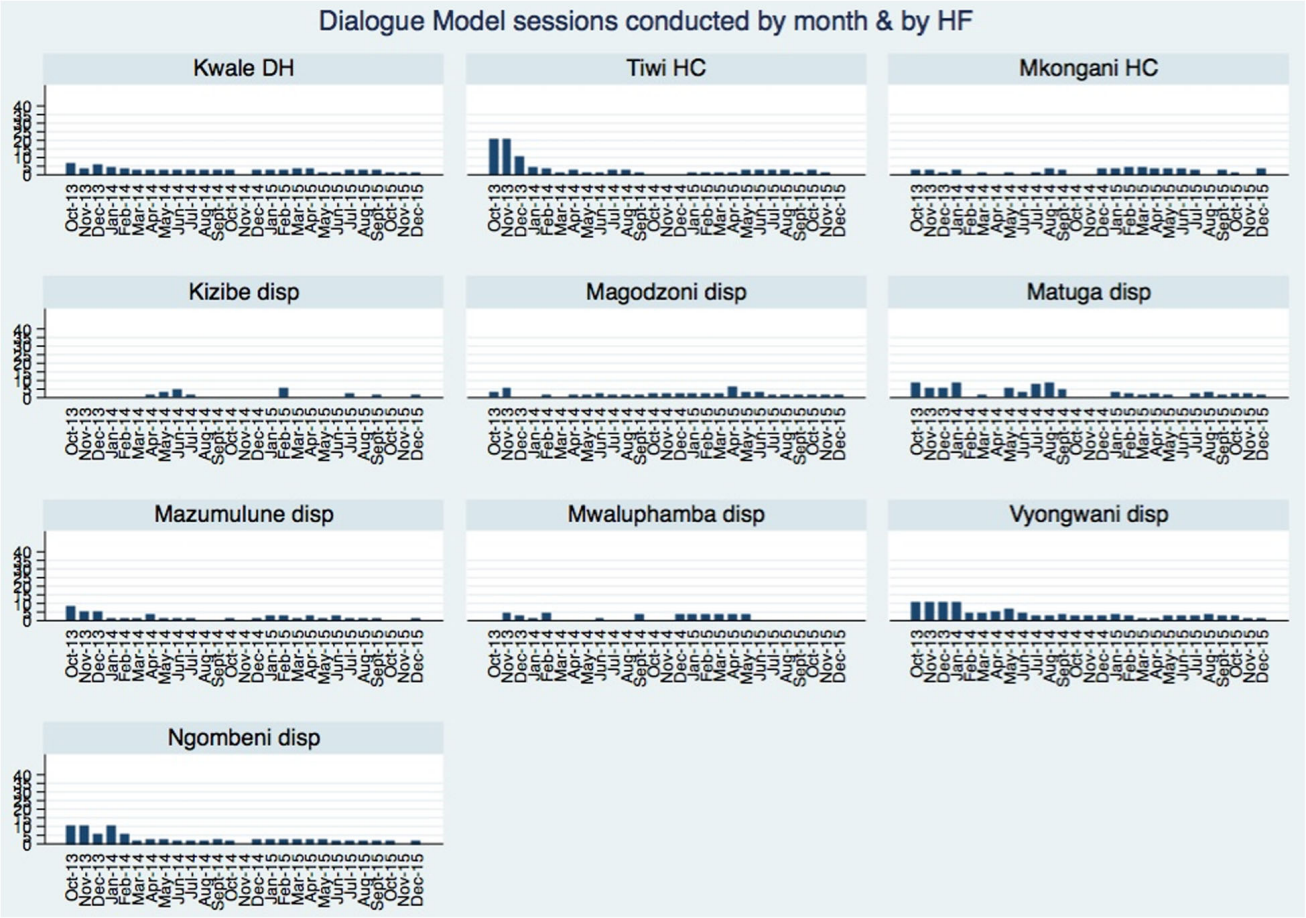
Number of Dialogue Model sessions conducted by month and by health facility in Matuga sub-county, Kwale

Overall, the median (IQR) number of DM sessions held per facility per month was 2 (1–3) with the range varying per facility (Table 2). In 27 months, Kizibe and Mwaluphamba dispensaries had 19 (70%) and 14 (52%) months respectively, during which no DM sessions were held in their associated CUs. Vyongwani dispensary held at least 1 DM session during all the months of intervention implementation. The highest number of DM sessions held in 1 month was 20 in the 2 CUs associated with Tiwi Rural Health Training Centre while the least number of DM sessions held in a facility was 4 in the CU associated with Mwaluphamba (Fig. 1).

**Table 2:** Dialogue Model sessions held among intervention health facilities (*N* = 10) and their associated community unities (*N* = 12) in Matuga sub-county, Kwale

Health facility name	Level of care*	Community unit(s)	Total DM held	Max no. DM/month	Median/IQR DM/month	Range DM/month
Kwale District Hospital	4	Chitsanze	60	6	2 (2–3)	0–5
Tiwi Rural Health Training Centre	3	Mwachema & Mkoyo	83	20	1 (1–2)	0–20
Mkongani Model Health Centre	3	Mkomba	46	4	2 (0–3)	0–4
Kizibe Dispensary	2	Kizibe	20	5	0 (0–1)	0–5
Magodzoni Dispensary	2	Simkumbe	46	6	1 (1–2)	0–6
Matuga Dispensary	2	Matuga	74	8	2 (1–5)	0–8
Mazumalume Dispensary	2	Mazumalume	43	8	1 (1–2)	0–8
Mwaluphamba Dispensary	2	Tserezani	33	4	0 (0–3)	0–4
Vyongwani Dispensary	2	Vyocuta	94	10	2 (2–4)	0–10
Ng’ombeni Dispensary	2	Mtamazide & 4Ms	69	10	2 (1–2)	0–10
Total			570	20	2 (1–3)	

*Refers to the previous levels of health care delivery in Kenya (1= Community, 2 = Dispensary, 3 = Health center, 4 = District/County referral hospital, 5 = National referral hospital

**Fig. 2:**
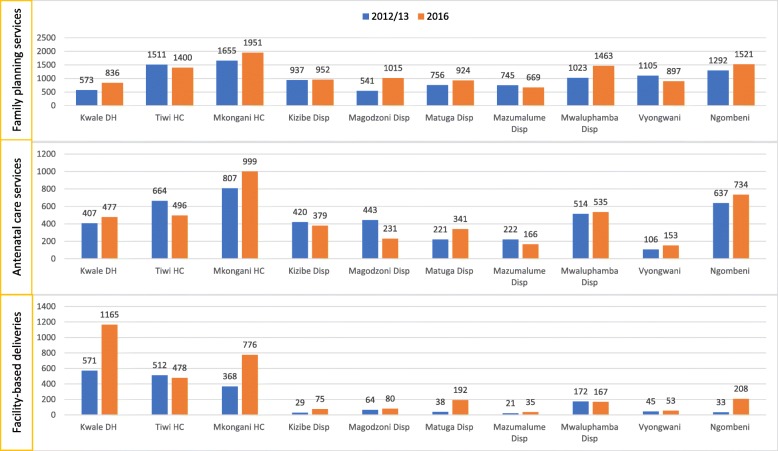
Change in uptake of family planning, antenatal care and facility-based deliveries pre- (October 2012 – September 2013) versus post- (January – December 2016) intervention implementation among intervention facilities

In the year before the intervention (October 2012–September 2013), the total number of WRA receiving FP commodities as reported by the 10 intervention facilities was 10,138 (Mean (M) = 1014, Standard Deviation (SD) = 381). The highest number was reported in Mkongani Model Health Centre at 1655 and the lowest in Magodzoni Dispensary at 541 (Fig. 2). Between January – December 2016, the total number of WRA receiving FP commodities was 11,628 (M = 1163, SD = 400). The highest number of WRA receiving FP commodities at this time point was reported in Mkongani at 1951 (18% increase) while the lowest was in Mazumalume dispensary at 669 which was a 10% decline. At 88%, Magodzoni dispensary reported the highest proportionate increase in number of WRA taking up FP services. Vyongwani dispensary reported the largest decline in FP uptake (19%) between these two time-points. Overall, FP uptake increased by 15% across the 10 intervention facilities.

Additionally, prior to the intervention (October 2012 – September 2013), the 10 intervention facilities reported a total of 4441 (M = 444, SD = 220) new ANC attendees, once again highest in Mkongani at 807 and lowest in Vyongwani at 106 (Fig. 2). Post-intervention implementation (January – December 2016), the total number of new ANC attendees reported by these facilities was slightly higher at 4511 (M = 451, SD = 264, a 2% increase. Just like at baseline, the highest and lowest numbers of new ANC attendees in 2016 were reported in Mkongani and Vyongwani at 999 and 153, respectively. The largest proportionate increase in uptake of ANC was reported in Matuga Dispensary (54%) while Magodzoni dispensary reported the largest decline (48%).

e total number of facility-based deliveries reported by the 10 intervention facilities at baseline was 1853 (M = 185, SD = 216). This number was highest for Kwale District Hospital at 571 and lowest for Mazumalume at 21. In 2016, the total number of facility-based deliveries had nearly doubled to 3229 (M = 323, SD = 384), again highest and lowest in Kwale and Mazumalume at 1165 and 35, respectively. Overall, uptake of facility-based deliveries increased by 74%. Ng’ombeni Dispensary reported the largest proportionate increase (530%) in facility-based deliveries while Mwaluphamba reported a 3% decline (Fig. 2).

Using a paired sample t-test, there were statistically significant increase in the number of WRA taking up FP services pre- (M = 1014; SD = 381) versus post- (M = 1163; SD = 400); t (18) = − 0.603, *P* = 0.04) as well as facility-based deliveries pre- (M = 185; SD = 216) versus post- (M = 323; SD = 384); t (14) = − 0.698, *P* = 0.03). The difference seen in number of new ANC attendance pre- (M = 444; SD = 220) versus post- (M = 451; SD = 264) was not statistically significant (t (18) = − 0.046, *P* = 0.43) [Table 3].

**Table 3:** Change in uptake of family planning, antenatal care and facility-based deliveries pre (Oct. 2012 – Sept. 2013) versus post (Jan. – Dec. 2016) Dialogue Model implementation in intervention health facilities (*N* = 10)

Health facility name	Family Planning				Antenatal Care				Facility-based Deliveries			
	2012/13	2016	Diff.	% Diff.	2012/13	2016	Diff.	% Diff.	2012/13	2016	Diff.	% Diff.
Kwale District Hospital	573	836	46	46	407	477	70	17	571	1165	594	104
Tiwi Rural Health Training Center	1511	1400	−7	−7	664	496	− 168	−25	512	478	−34	−7
Mkongani Model Health Center	1655	1951	18	18	807	999	192	24	368	776	408	111
Kizibe Dispensary	937	952	2	2	420	379	−41	−10	29	75	46	159
Magodzoni Dispensary	541	1015	88	88	443	231	−212	−48	64	80	16	25
Matuga Dispensary	756	924	22	22	221	341	120	54	38	192	154	405
Mazumalume Dispensary	745	669	−10	−10	222	166	−56	−25	21	35	14	67
Mwaluphamba Dispensary	1023	1463	43	43	514	535	21	4	172	167	−5	−3
Vyongwani Dispensary	1105	897	−19	−19	106	153	47	44	45	53	8	18
Ng’ombeni Dispensary	1292	1521	18	18	637	734	97	15	33	208	175	530
Total	10,138	11,628	15	15	4441	4511	70	2	1853	3229	1376	75
Mean	1014	1163			444	451			185	323		
Standard deviation	381	400			220	264			216	384		
T-statistic		−0.603				−0.046				−0.698		
Degrees of freedom (dF)		18				18				14		
*P*-value (1-tail)		0.04				0.43				0.03		

To understand the effect of concurrent interventions in the area on the outcomes of interest, similar comparisons pre- versus post-intervention implementation were conducted for the 10 remaining facilities that did not receive the intervention. Amongst these, there was an 8, 11 and 8% change in uptake of FP, ANC and facility-based deliveries, respectively (Table 4). However, these differences were not statistically significant for either of the outcomes, including uptake of FP services pre- (M = 720; SD = 259) versus post- (M = 776; SD = 396); t (18) = − 2.657, *P* = 0.33), new ANC attendance pre- (M = 120; SD = 86) versus post- (M = 134; SD = 87); t (18) = − 0.246, *P* = 0.27) as well as facility-based deliveries pre (M = 65; SD = 90) versus post- (M = 70; SD = 79); t (18) = − 0.097, P = 0.33).

**Table 4:** Change in uptake of family planning, antenatal care and facility-based deliveries pre (Oct. 2012 – Sept. 2013) versus post (Jan. – Dec. 2016) Dialogue Model implementation in non-intervention health facilities (*N* = 10)

Health facility name	Family Planning				Antenatal Care				Facility-based Deliveries			
	2012/13	2016	Diff.	% Diff.	2012/13	2016	Diff.	% Diff.	2012/13	2016	Diff.	% Diff.
Mwapala Dispensary	843	631	−212	−25	60	178	118	197	26	31	118	197
Msulwa Dispensary	704	426	−278	−40	140	79	−61	−44	80	33	−61	−44
Shimba Hills Health Center	1083	870	− 213	−20	280	212	−68	−24	297	255	−68	−24
Lukore Dispensary	652	483	− 169	−26	109	55	−54	−50	14	29	−54	−50
Kiteje Dispensary	423	561	138	33	32	139	107	334	7	48	107	334
Mkundi Dispensary	746	1126	380	51	108	95	−13	−12	2	29	−13	−12
Kibuyuni Dispensary	621	534	−87	−14	43	47	4	9	15	30	4	9
Mwaluvanga Dispensary	644	1674	1030	160	29	36	7	24	11	8	7	24
Mbuguni Dispensary	324	468	144	44	165	188	23	14	113	181	23	14
Waa Dispensary	1156	985	− 171	−15	235	307	72	31	81	54	72	31
Total	7196	7758	562	8	1201	1336	135	11	646	698	52	8
Mean	720	776			120	134			65	70		
Standard deviation	259	396			86	87			90	79		
T-statistic		−2.66				−0.25				−0.10		
Degrees of freedom (dF)		18				18				18		
P-value (1-tail)		0.33				0.27				0.33		

## Discussion

In this before-after pragmatic study, we found a significant increase in the uptake of FP services and facility-based deliveries in facilities associated with CUs where we implemented a structured, community-participatory intervention targeted at improving uptake of these services in Kwale County, Kenya. We also found a slight increase in the uptake of ANC services, but this was not statistically significant. Our findings suggest that implementing a structured, community-participatory intervention could contribute to enhancing uptake of select MCH services in a rural Kenyan setting.

It is worth noting that the enhanced uptake of FP services and facility-based delivery reported in this study corresponds with high utilization rates for these MCH services that we have previously reported in this setting from findings of a household survey [40, 41]. In our previous work, we reported a high CPR of 54%, low unmet need for FP at 16% and a facility-based delivery rate of 78%. These findings also follow a general trend seen in recent KDH surveys that show an overall increase in uptake of FP and facility-based delivery in this setting [42–44].

Our findings also reflect potential gains derived from decentralization of health services in Kenya. This decentralization has made available resources and devolved decision-making to a local system of governance that is better placed at identifying locally-responsive solutions to public health issues [45]. In this regard, the County Government of Kwale has made significant investments aimed at strengthening the local health system so as to create an enabling environment to ensure uptake and utilization of MCH services [46–48]. While the bulk of these resources have gone into improving supply-side factors like putting up the necessary infrastructure, employing and enhancing the capacity of HCWs and improving the supply chain for medical commodities; a significant proportion has also been invested in building demand for health services through community-led initiatives.

The success of community-participatory approaches for MCH interventions hinges on the participatory model chosen [24, 49, 50]. Community-organized actions employ a model that relies on the intrinsic motivation of community members to develop and implement the interventions, in contrast with extrinsically-induced community participation that is driven primarily by external stakeholders. A key aspect of the DM intervention was that it was fully community-led without any active external influence from the project team, save for occasional supportive supervision visits. Prior to intervention implementation, we trained CHVs on how to effectively conduct DM sessions. After the training, we relied on them to obtain necessary buy-in from relevant community gatekeepers, organize and mobilize session participants, spearhead sessions and take responsibility for the final action plans developed.

Our findings need to be interpreted within the context of several limitations. Using a before-after pragmatic study design did not allow us to fully account for the potential confounding effect of concurrent interventions. Principally, just before we began implementing the DM sessions, the Government of Kenya enacted a policy of free maternity care in all public health facilities. Within several months of implementing the policy, the proportion of facility-based deliveries reported nationally had increased significantly [51]. We accounted for this limitation by conducting a similar comparison of outcomes in the remaining 10 non-intervention facilities which showed a slight, non-significant change in uptake of the same MCH services pre-versus post-intervention implementation.

In our case, non-intervention facilities served a small catchment population and were generally considered to be low-volume. For this reason, they were not prioritized in the initial setting up of CUs and selection/training of CHVs. Regardless, implementation of the free maternity care policy was rolled out countrywide without consideration of the facility workload. In fact, each facility was reimbursed a standard fee by the Kenyan government for every delivery reported and these resources were used to improve local service delivery. It was therefore, in the best interest of each facility to mobilize for additional deliveries. To further illustrate this point, while intervention facilities reported ~ 75% increase in uptake of facility-based deliveries pre-versus post-implementation, there was only a marginal increase in non-intervention facilities (8%), suggesting an effect over and above that of the free maternity policy. Finally, this policy did not target uptake of FP services and cannot account for the changes in FP uptake reported in the intervention facilities.

To our knowledge, apart from the national free maternity policy, there were no other interventions targeting health systems strengthening for MCH services in Matuga sub-county, Kwale during the period of DM implementation. Additionally, the County Government of Kwale did not establish any additional CUs associated with intervention and/or non-intervention facilities during this time. The scope of work for CHVs did not also change in the course of intervention implementation. As part of their community activities, CHVs typically provide referrals, and in some instances, physically escort WRA, including pregnant mothers, to the nearest facility within their jurisdiction.

The MOMI project also aimed to strengthen the capacity of county and sub-county health management teams to conduct supportive supervision and provided specific on-job training for HCWs on emergency obstetric care and FP services. There was also an element of service delivery expansion given that some of the intervention facilities were capacitated to better offer services that they were not capable of previously offering. Since they were implemented in the same intervention facilities, the effect of these additional interventions was most likely complementary to that of the DM intervention.

Additionally, we did not collect individual-level data on the MCH outcomes of interest to demonstrate individual-level behavior change. Data collection was at an aggregate facility level and as such, we were unable to verify whether the women reported as taking up these services had actually attended a DM session that influenced their choice. However, due to the proximity of CUs where we implemented the intervention to the health facility and the intensity of intervention implementation, it is less likely that the WRA who eventually accessed these services could have come from other communities apart from those associated with the intervention facilities. The rates of reporting into the DHIS-2 by both intervention and non-intervention facilities ranged from 90 to 100%. Additionally, the rates of in- and out-migration from this rural community are generally low and we can assume, with some level of certainty, that most WRA remained in their communities during the period of the study and likely interacted with CHVs implementing the DM intervention.

We also report absolute numbers of women receiving FP, ANC services and those who delivered in a health facility as the outcomes of interest. Since these numbers could have changed over time, the ideal outcome indicators would have been the proportion of women initiating FP, newly-attending ANC and delivering in a health facility out of the total number of WRA or number of pregnancies. Without accurate denominator information, a population-based survey would have been a better way of measuring this change which would also have allowed measurement of the level of exposure to the DM intervention. In this case, unlike the numerator data which was abstracted from the DHIS-2, the denominator in our case is an estimate derived from the annual workplan and would not accurately capture the information intended.

Finally, we sampled the villages and participants to DM sessions purposively, mainly at the convenience of the CHVs organizing the session. This approach may have introduced a selection bias in the manner that our intervention was implemented. Our approach was pragmatic given that we aimed to assess the effect of an intervention that relied on the presence of an active CU to be implemented. We also wanted to have an intervention that could be implemented within a real-world setting and represent the reality on the ground. It was therefore impractical to set up separate CUs that could not be maintained at the end of the project. We also aimed for the intervention being fully community-led with minimal external influence apart from occasional supportive supervision. It was informed by previous work that has demonstrated greater efficacy for participatory approach models that are fully community-led. Our study design accounted for the potential selection bias by comparing outcomes pre- versus post-intervention implementation in the same sample of facilities thereby reducing any inter-facility variability.

## Conclusion

We found a significant increase in the uptake of FP services and facility-based deliveries in facilities associated with CUs where we implemented a structured, community-participatory intervention targeting to improve uptake of these services. These findings reflect a need for programs to include community participatory approaches as a key component as it enables them to implement interventions that are culturally-sensitive and locally-responsive. Ultimately, it is a useful approach in addressing demand-side factors for enhanced uptake of MCH services by providing communities with a stake in influencing their health outcomes.

## Additional file

Additional file 1: Study-specific procedures for conducting a dialogue model session. This file contains procedures developed to guide in organizing and moderating a dialogue model session. (DOCX 104 kb)

## Abbreviations

ANC: Antenatal care
CHEW: Community health extension worker
CHV: Community health volunteer
CI: Confidence interval
CPR: Contraceptive prevalence rate
CU: Community unit
DHIS-2: District health information system-2
DM: Dialogue Model
FP: Family planning
HCW: Health care worker
IQR: Interquartile range
KDHS: Kenya Demographic and Health Survey
MCH: Maternal and child health
MoH: Ministry of Health
MOMI: *Missed opportunities in maternal and infant health* project
WRA: Women of reproductive age
