# Supplementary material for: “Our voices matter”: a before-after assessment of the effect of a community-participatory intervention to promote uptake of maternal and child health services in Kwale, Kenya
Source: BMC Health Serv Res. 2018 Dec 4;18:938. doi: 10.1186/s12913-018-3739-9 (PMC6280535; doi:10.1186/s12913-018-3739-9)
Supplement: Supplementary file 1 — Study-specific procedures for conducting a dialogue model session. This file contains procedures developed to guide in organizing and moderating a dialogue model session. (DOCX 104 kb) [file 12913_2018_3739_MOESM1_ESM.docx]

**MOMI Project: Study Specific Procedure (SSP 001)**

**Title: Procedure for conducting Dialogue Model sessions**

________________________________________________________________

Version: 01 Date: 15/OCT /2013

________________________________________________________________

Authorization:

These signatures below constitute the approval of the SSP **001** version 0**1** dated **15/OCT/2013** and provide assurance that the study will be conducted according to these procedures.

Principal Investigator:

Signed: _____________________

Full name: **Prof. Peter Gichangi**

Date :( DD/MM/YYYY) _____________________

Project Manager/Doctor:

Signed: _____________________

Full name: **Dr. Vernon Mochache Oyaro**

Date: (DD/MM/YYYY) _____________________

________________________________________________________________

Author: **Dr. Vernon Mochache**

Effective date: 15^TH^ OCT 2013

***________________________________________________________________***

**INDEX**

1. Introduction
2. Applicable to
3. Abbreviations
4. Equipment and Materials
5. Procedure
6. Appendices
7. Revision History
8. **Introduction**

This SSP describes the procedures used in conducting Dialogue Model meetings for the **M**issed **O**pportunities in **M**aternal and **I**nfant health (MOMI) project.

The Dialogue Model seeks to promote a deeper understanding of the efforts of communities, households and individuals in staying healthy in order that external interventions can be more appropriate and relevant. This would ensure a negotiated decision on vital interventions for health and avoids displacing people’s efforts with short-term unsustainable actions.

This organized approach can be applied at entry, at feedback, at planning and at implementation of planned interventions towards sustainable behavior change, involving the service delivery system, the health workers (HWs) and target communities as partners for lasting change. The Dialogue Model is built on the principles of partnership, negotiation; problem-based learning (PBL) as well as building healing communities.

This strategy is being used to facilitate the uptake of postpartum family planning (PPFP) in Matuga Sub-County, Kwale County. The MOMI-Kenya team will train Dialogue Model facilitators from among CHEWs in the MOMI-mapped health facilities as well as selected CHWs.

1. **Applicable to**

- MOMI-Kenya team members
- MOMI-selected health facilities sites
- MOMI-selected Community Units (3-4)

1. **Abbreviations**

CHWs Community Health Workers

CHEWs Community Health Extension Workers

CUs Community Units

DM Dialogue Model

HCWs Health Care Workers

ICRH-K International Centre for Reproductive Health - Kenya

MCH/FP Maternal and Child Health and Family Planning

MNH Maternal and Newborn Health

MOMI Missed Opportunities in Maternal and Infant health

PBL Problem-based learning

1. **Equipment and materials**

- Health informational poster
- Notepad
- Pen

1. **Preparation for Dialogue Model session – Health facility**

- The preferred department to conduct DM sessions is the MCH/FP Clinic
- The CHEW will be responsible for organizing and conducting or delegating the conduct of DM sessions
- The HCW responsible will select the starter (picture, skit or role-play) in preparation for the session
- They will organize the sitting arrangement so as to allow for ideal conduct of the DM session

1. **Preparation for Dialogue Model session – Community**

- The responsible CHW will organize separate DM sessions that are gender and age-specific and inform a MOMI-Kenya team member. They will also occasionally organize mixed DM sessions
- The CHW will set a date and venue for the DM session and contact the local administrator (chief/village elder) to inform him of the planned session
- The responsible CHW will determine the target participants by asking community members “Who would you like to bring together to share ideas and opinions on postpartum family planning?”
- The CHW will also inform the health facility in good time so that they can organize for a HCW to be present to answer health-related issues raised during the session
- They will also inform the other CHWs so as to mobilize community members for the DM session. The total number of community members mobilized should not exceed 40 per session

1. **Required skills**

The person chosen to moderate over DM sessions will be adapted to the age and gender of the session’s participants. During the dialogue process, the moderator should:

- Use Open-ended and probing questions
- Affirmation/appreciation
- Reflective listening/paraphrasing
- Summarizing
- Remain neutral
- Ensure that they are able to maintain eye contact with all the participants at all times during the DM session
- Maintain group control since some participants are likely to dominate. Make sure the silent ones are also involved.
- Let the conversation build from the known to the unknown

1. **Procedures**
   - Participants will receive an explanation of the session as a group and any that is not comfortable will be given an opportunity to leave. The remaining participants will provide oral consent to participate as a group
   - The Dialogue Model process will be initiated by a dialogue stimulator code (starter) such as a song, role-play, case study, informational poster or story.
   - This is intended to stimulate discussion, focusing on the issue of PPFP. This starter will be **s**hort, **s**ensitive to local culture, **s**imple, **s**pecific, posing a **s**ingle problem without providing a **s**olution and adapted to the target audience
   - The Dialogue Model moderator will follow the steps outlined (Appendix 9.1) which includes a series of questions to:
     - Identify and define the issues around PPFP and confirm its relevance to the audience, by asking open-ended question such as “**What did you see or hear?**” “**What was the problem posed?**” and “**Does the problem occur in this community?**” This is illustrated by individual testimonies of actual experiences with the problem in terms of occurrence and consequences. This step will get people talking and enables them identify and name the issue from their own perspective and to emotionally own the problem as well as begin to reflect on the needed improvement
     - Identify current actions (alternatives) to address the issue and the extent to which they achieve desired results. The question may be asked as to “**Why does the problem persist?**” in spite of current efforts. This step will promote analysis of the causes of the current issue, reaching a consensus that the current situation can be improved, and a better future is possible
     - Identify new actions (options) necessary to solve the issue of PPFP from the perspective of the community as well as the perspective of the HWs. Through brainstorming a list of options will be generated and appraised in terms of effectiveness and feasibility. This step benefits from positive deviants in the community. Those willing to consider or might have tried new ways since it is easier for people to learn from their peers, people who share their realities. A set of doable options is agreed on
     - Generate commitment by asking the people to consider and list the consequences of taking the recommended action or not taking. Hopefully, the consequences of action are more, to establish the benefits of action. “**What do you think will be the results of carrying out the recommended action?”**
   - Having confirmed the importance and urgency of action to be taken, the group will then prepare a plan of action detailing what will be done, by whom, when and with what resources
   - The facilitator will keep a written record of the meeting attendees as well as the agreed upon plan of action
   - Other PNC issues raised during such meetings will also be captured and recorded
   - The MOMI-Kenya team will assess implementation of the plan of action during facilitative supervision visits
2. **Appendices**

Appendix 9.1: **Steps in moderating a Dialogue Model session**

| **STEP** | **DESCRIPTION** | **KEY QUESTIONS** |
| --- | --- | --- |
| ***1.***  Listen for issues and efforts | **Problem and capacity identification**  -Recognize efforts and desired outcomes  -Probe for current alternatives, **often hidden actions**, if new action not agreed with | ***What*** did you see or hear  ***What is the problem***  Does it happen in your community?  ***(give personal or community examples). What are the current concerns and how are they affected by it?*** |
| 2.  Listen for why | **Problem and capacity Analysis**  -Identifying reasons or causal explanations  History, track record, effort and resources available, obstacles and opportunities | ***Why*** does the problem happen?  What are the reasons for doing only the things you do  ***(not some of the preferred ones)*** |
| **3**  Listen for future they want & action | **Options identification**  -Searching together for possible solutions to improve the current situation  -Identify workable options actions to improve the current situation  **Options appraisal and selection of best:**  -effectiveness, feasibility based on own resources, with tangible results, can scale up | **What** is the future we desire?  **What** can we do to solve the problem/situation, to achieve the desired future? (add the recommended actions on the list, if not mentioned or summarize from their contributions) **Which options from the agreed list should be adopted** |
| **4.**  Listen to why action | **Commitment establishment** by summarizing agreement, articulating consequences, these become indicators for monitoring progress and outcome | **What do you think would happen if you do or do not take the agreed action** |
| **5.**  Listen & plan action with them | **Action plan**  -mapping out what has to be done –identify activities which the community can undertake by themselves with minimal assistance, but which will have tangible results in a relatively short period of time | ***When***, can we take action and ***who,*** is responsible |
| ***6.***  Assess | **Assessment and feedback** based on information (note modifications, compliance or rejection). Feedback, celebrate results, recognize excellence and re-plan, making necessary adjustments. | How will we know our progress, who has made it possible and how can we sustain change? |

1. **References**

- “MOMI Project Intervention Protocol” version 1.1 dated 28^th^ September 2013
- The Dialogue Model: Community Integrated Management of Childhood Illnesses

1. **Revision history**

| Date Reviewed | Date Revised | Replacement SSP # | Initials |
| --- | --- | --- | --- |
|  |  |  |  |
|  |  |  |  |
